# The positive effect of spermidine in older adults suffering from dementia: First results of a 3-month trial

**DOI:** 10.1007/s00508-020-01758-y

**Published:** 2020-11-19

**Authors:** Thomas Pekar, Katharina Bruckner, Susanne Pauschenwein-Frantsich, Anna Gschaider, Martina Oppliger, Julia Willesberger, Petra Ungersbäck, Aribert Wendzel, Alexandra Kremer, Walter Flak, Felix Wantke, Reinhart Jarisch

**Affiliations:** 1 Biomedical Science, University of Applied Sciences Wiener Neustadt, Johannes-Gutenberg-Str. 3, 2700 Wiener Neustadt, Austria; 2 Gepflegt Wohnen GmbH, 182, 8412 Allerheiligen bei Wildon, Austria; 3 Privatklinik Rudolfinerhaus GmbH, Billrothstr. 78, 1190 Vienna, Austria; 4 Federal Office for Viticulture, Gölbeszeile 1, 7000 Eisenstadt, Austria; 5 FAZ Floridsdorfer Allergiezentrum, Pius-Parsch-Platz 1/3, 1210 Vienna, Austria

**Keywords:** Dementia, Alzheimerʼs disease, Spermidine, CERAD, Kognitive performance

## Abstract

The worldwide prevalence of dementia is estimated at 35.6 million and will rise to 115 million by 2050. There is therefore an urgent need for well-founded dementia diagnostics and well-researched therapeutic options. Previous studies have highlighted that spermidine has the ability to trigger the important process of dissolving amyloid-beta plaques by autophagy. They also confirmed that nutritional intervention with the natural polyamine spermidine can prevent memory loss in aging model organisms. This multicentric double-blind preliminary study focused on the effect of oral spermidine supplementation on older adults’ cognitive performance. Memory tests were carried out on 85 subjects aged between 60 and 96 years in 6 nursing homes in Styria. Blood samples were taken for the determination of spermidine concentration and measurement of metabolic parameters. The results demonstrated a clear correlation between the intake of spermidine and the improvement in cognitive performance in subjects with mild and moderate dementia in the group treated with the higher spermidine dosage. The most substantial improvement in test performance was found in the group of subjects with mild dementia with an increase of 2.23 points (*p* = 0.026) in the mini mental state examination (MMSE) and 1.99 (*p* = 0.47) in phonematic fluidity. By comparison, the group which had a lower spermidine intake showed consistent or declining cognitive performance.

## Introduction

Age-associated disorders, in particular dementia, have become increasingly more prevalent in the media and politics in the last few years. The numbers speak for themselves—according to the Austrian Dementia Report of 2014 the number of people in Austria who will be suffering from dementia in 2050 is estimated at 262,200. Compared to 90,500 people in 2000, this represents a threefold increase in neurocognitively impaired people in 50 years [1]. The worldwide prevalence is currently estimated at 35.6 million and will rise to 115 million by 2050 [2]. Much of the increase will take place in developing countries and 58% of people with dementia live in low and middle income countries, but by 2050 it will be 68%. The fastest growth in aging population occurs in China, India and their South Asian and Western neighbors [3]. In a recent study in which the effects of the psychotropic drugs selective serotonin reuptake inhibitors (SSRI), trazodone and atypical neuroleptics/benzidiazepines on cognition and behavioral symptoms were investigated, no significant effects could be found [4]. Therefore, there is an urgent need for well-founded dementia diagnostics and well-researched therapeutic options that not only mitigate the process of neurocognitive loss of function or treat comorbidities but also start at the onset of the disease and at best alleviate, reverse or even cure the disease.

The preliminary results already showed a significant correlation between the spermidine levels in serum and memory performance [5].

### Spermidine and its effects

Increased concentrations of spermidine can positively influence cardiovascular and tumor-related diseases and inflammation through the essential contribution to cell homeostasis, cell growth, cell proliferation, tissue regeneration and antioxidant effects [6]. The effect of spermidine on neurocognitive impairment has been investigated in particular in the fruit fly *Drosophila melanogaster*. Numerous positive effects of this polyamine on memory performance with respect to age-related memory loss were observed. Spermidine has the ability to trigger the important process of autophagy by the dissolution of amyloid-beta plaques. Fruit flies supplemented with spermidine showed significant improvements in short-term and medium-term memory compared to flies of the same age which had not received the polyamine [7].

### Spermidine levels in food

The highest spermidine content was found in whole grain and wheat germs at a level of 243 mg/kg. Legumes, such as soybeans with 207 mg/kg spermidine, are the next largest. The levels found in cheese varies greatly, depending on the length of the ripening period. High spermidine levels are also found in mushrooms and chicken liver. Spermidine is also contained in fruits, such as mango, in semen, and especially in red wine [8–10]. The average daily intake of spermidine has been described as approximately 10 mg per person in developed countries [11].

### Research question and aim

The question of what impact spermidine has on the cognitive performance of older people in connection with dementia will be answered in this article. The aim of the research project was to show a possible improvement of neurocognitive functions when spermidine is taken orally. A concurrent low-dose group serves as a comparison. Additional metabolic functions were tested and correlated with cognitive performance and spermidine concentration.

## Experimental section

The design of the study was randomized, two-group, double-blind, multicentric and longitudinal.

In total, 92 older individuals (60–96 years of age, 70 female and 22 male fluent German speakers) participated in the study. Participants were recruited via the directors of nursing in six rest homes of the “Gepflegt Wohnen” group in Styria, Austria. People aged between 60 and 100 years were eligible for participation in the study. Furthermore, they had to not only take part in both the CERAD-Plus test and venepuncture but also continue their previous medication. Exclusion criteria comprised receiving antidementia medication, changing their previous medication, withdrawal by choice or participation in another study.

Written informed consent was obtained from all participants in accordance with the Ethics Committee of the Medical University of Graz, Austria (30-280 ex 17/18). For those who were not able to understand the study, relatives or legal guardians provided consent.

In the implementation of the study, the 92 subjects were divided into two random groups. One group received a grain roll with wheat germ (Schalkmühle, Ilz, Austria; 1075 mg/kg spermidine) for breakfast 6 times a week (roll A). Each roll A contained 3.3 mg of spermidine after baking. To scrutinize the success of spermidine, the second group received rolls baked with wheat bran (Schafler Mühle, Feistritz, Austria; 115 mg/kg spermidine) instead of wheat germ (roll B). Each finished roll B contained 1.9 mg of spermidine. Both the wheat germ and the wheat bran were added to the dough mixture during preparation.

On average, the subjects ate 68 rolls during the 3 months of the study. The maximum number of rolls eaten was 79 and the minimum zero. This amounts to an increase in the average daily spermidine intake of approximately 35% in the group that received roll A and 20% in the group that received roll B.

The monitoring of other food sources for spermidine was neglected because all subjects had the same choice of menus.

To test the cognitive performance of the subjects the CERAD-Plus test was implemented before the start and by the end of the study. The test was created by an American research association called “The Consortium to Establish a Registry for Alzheimer’s Disease”, in 1986. It serves as a database for patients with Alzheimer’s. The Memory Clinic of the University Hospital in Basel published a German version of this test in 1998 (www.memoryclinic.ch). The CERAD-Plus test involves seven individual examinations to audit orientation, language, constructive practice and memory.

The seven tests are:

- Verbal fluency
- Boston naming test
- Mini mental state examination (MMSE)
- Learn, recall and recognize a word list
- Sign and recall figures
- Trail making test A and B
- Phonemic fluid

The CERAD-Plus questionnaire is highly objective, reliable, economical and extremely easy to conduct [12]. The z‑value was used for group comparisons. The z‑value is a standardized, calculated value that has been corrected for age, gender and years of education. Thus, all test results were standardized in order to improve comparison of the results and to ascertain the proximity of the achieved values from the normal range. Values from +5 to −5 can be attained, 0 being the calculated value for a normal test person. Numbers in the minus range show that the respondent’s results are below the normal range and values above 0 show better results than the average for a person of the same age, education level and gender [13]. The CERAD total score was used for comparison of the overall cognitive performance between the intervention groups. The score was calculated analogous to the description by Chandler et al. [14].

For the quantitative determination of spermidine levels, a competitive ELISA kit (abx585001; Abbexa, Cambridge, UK) was used. The tests were carried out in accordance with the manufacturer’s instructions and the rules for good laboratory practice. The concentration of all available samples was measured at four different times and four standards with concentrations of 12.5–200 ng/ml were used to calculate the sample concentration. The intra-assay and interassay variability of the used batch were <10% and <12%, respectively. Laboratory diagnostics are essential if a neurocognitive disease is suspected. Thus, metabolic performance of the human organism was tested. In this study, thyroid function, iron storage and vitamin B12 and folic acid metabolism were tested and correlated with spermidine concentrations and cognitive performance. The thyroid-stimulating hormone (TSH), ferritin, vitamin B12 and folic acid were measured on Roche’s Cobas e411 (Roche Diagnostics GmbH, Mannheim, Deutschland) via electrochemiluminescent immunoassay. The determination of the spermidine content in the raw materials and finished bakery products was carried out by LVA GmbH (Klosterneuburg, Austria) using HPLC according to the methods MUVA-MET021 and to ASU L10.00-5:1992-12 mod.

The calculation of needed cases to show an effect (case number estimation) was performed with G*Power Vers. 3.1.9.2 (University of Düsseldorf, Düsseldorf, Germany). The calculation was based on a target difference of 3 points in MMSE and dz = 0.35. An a priori sample size of 74 participants (35 per treatment arm) was estimated to provide 67% power with alpha equal to 0.05.

Statistical analyses were carried out with the SPSS 25 statistical package (PASW, SPSS; IBM, Armonk, NY, USA). The relationship was analyzed using Pearson correlation and Kendall rank correlation coefficient tau‑b. Wilcoxon and Mann-Whitney’s U‑test was applied for two-group comparison. The level of significance was set at a *p*-value of less than 0.05. A correction for multiple testing was applied when performing the post hoc tests. The alpha-level was adjusted using Bonferroni correction: 0.05/3 = 0.0167. A *p*-value of <0.0167 was therefore assumed to be significant for the interpretation of the results.

## Results

### Participants

**Fig. 1:**
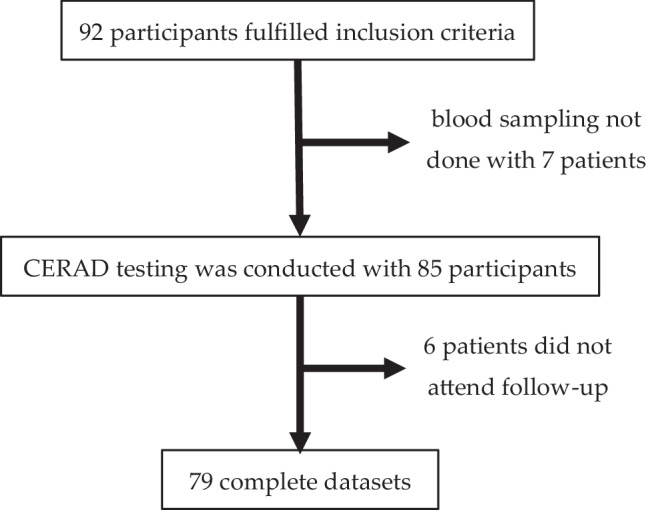
Study flowchart

A total of 85 participants were used for further analyses (Fig. 1).

The average age of the participants was 83.1 years (interquartile range, IQR 79–89.5 years). In group A there were 33 women and 10 men, and in group B 34 women and 8 men.

For further analyses, the subjects were divided into the following 4 groups based on the results of the mini-mental test (MMSE):

- No dementia (MMSE >26)
- Mild dementia (MMSE = 21–26)
- Moderate dementia (MMSE = 12–20)
- Severe dementia (MMSE <12)

The Mini-Mental status is a partial test, whereby the subjects can reach 0 to 30 points [15]. Table 1 demonstrates the final frequencies in the individual subcohorts. The mean MMS value in group A was 24.0 and in group B 24.5.

**Table 1:** Number of cases in subcohorts divided in high (A) and low (B) dosage spermidine intake group and gender (*m* male, *f* female)

	Group A		Group B		Total
	m	f	m	f	
No dementia	3	9	3	8	23
Mild dementia	3	15	2	15	35
Moderate dementia	2	6	3	11	22
Severe dementia	2	3	–	–	5
Total	10	33	8	34	85

The examination of a potential influence by the individual assessors of the CERAD-Plus test revealed no significant correlation between the interviewer and the result of the test (*p* = 0.913).

### Spermidine levels

In the case of bakery products (rolls A and B), the actual spermidine content was determined. Analysis of the grain roll A which was given to group A showed a spermidine content of 3.3 mg/piece and the roll which was given to group B (roll B) 1.9 mg/piece. On average, the subjects ate 68 rolls during the 3 months of the study. In the first month 24.6 rolls were eaten, in the second month 22.0 and in the third month 21.4. The participants in group A therefore consumed an additional 238 mg of spermidine on average over the duration of the study, while group B consumed an additional 137 mg of spermidine on average.

**Fig. 2:**
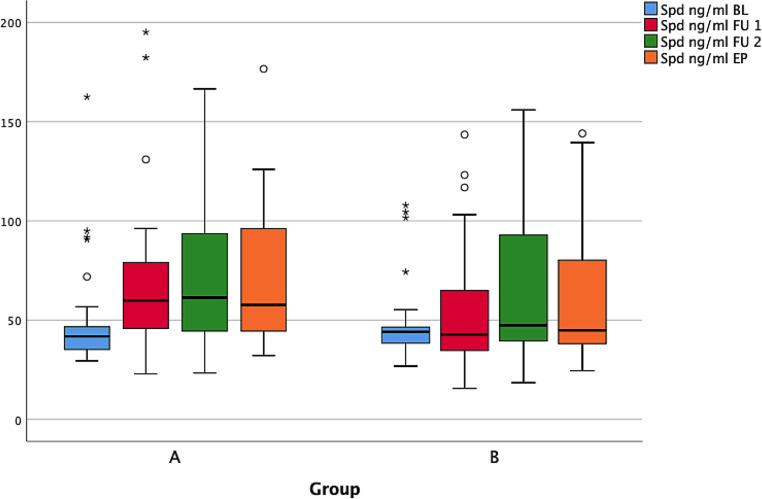
Serum spermidine course in groups A and B; measurement times: baseline, follow-up 1 after 1 month, follow-up 2 after 2 months and endpoint after 3 months. *Spd* spermidine, *BL* baseline, *FU 1* follow-up 1 month, *FU 2* follow-up 2 months, *EP* endpoint (o cases with values between 1.5 and 3 times the IQ range; * cases with values more than 3 times the IQ range)

Analyses of the ingredients and finished baked goods showed that spermidine is heat-sensitive, contrary to an earlier published statement [16]. In the present case, the quotient is about 3.4 between spermidine used (roll A: 149.4 mg/kg; roll B: 98.8 mg/kg) and the concentration in the finished baked goods (roll A: 47.1 mg/kg; roll B: 26.5 mg/kg). The median of the blood-spermidine level in group A was 41.65 ng/ml at the beginning of the study which increased to 58.20 ng/ml, 57.20 ng/ml and lastly 59.40 ng/ml. At the beginning of the study the spermidine level of group B was 44.70 ng/ml. In the following 3 months no real increase was noted (Fig. 2). The medians were 41.38 ng/ml, 44.71 ng/ml and 44.25 ng/ml. The significant *p*-value for group A was <0.001 and for group B 0.106.

### Memory performance

**Fig. 3:**
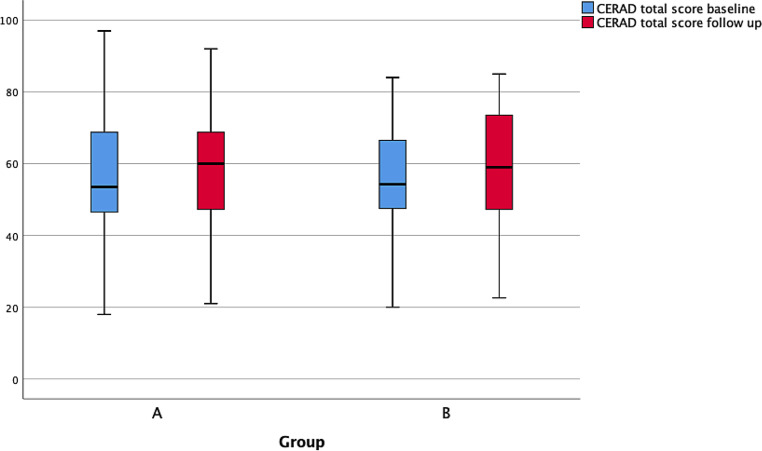
CERAD total scores in groups A and B; measurement times: baseline and follow-up after 3 months

The comparison of the CERAD-Plus total score showed an increase in both intervention groups. In detail, the high-dose group improved by 6.25 points (p = 0.030) and the low dose group by 4.00 points (p = 0.041) (Fig. 3).

**Fig. 4:**
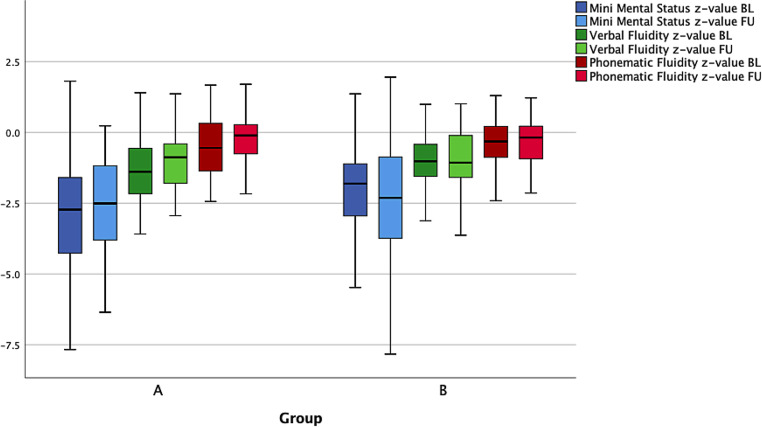
Comparison of CERAD-Plus test items; measurement times: baseline and follow-up after 3 months

The evaluation of the CERAD-Plus test showed an increase of the mean z‑scores for the items “Mini Mental Status” (−3.32 vs. −2.82), verbal fluidity (−1.39 vs. −0.97) and phonematic fluidity (−0.44 vs. −0.12) in group A between baseline (the measurement at the beginning of the study) and follow-up (measurement after 3 months). In group B the respective median remained unchanged or showed a slight decrease (Fig. 4).

The most substantial improvement in test performance for group A was found in the group of subjects with mild dementia with an increase of 2.23 (*p* = 0.026) in Mini Mental test and 1.99 (*p* = 0.47) in phonematic fluidity. Correlation between the individual CERAD items and the spermidine concentration was analyzed at the time of preintervention. Considering the entire cohort and the dementia groups mild dementia, moderate dementia and severe dementia, no significant positive correlations were observed but for certain CERAD items, significant negative correlations were seen.

In the group without cognitive impairment (“no dementia”) only positive significant correlations were found. Significant correlations were revealed with word list recognition discriminability raw value (*p* = 0.047), verbal fluency animals raw value (*p* = 0.042), Mini Mental Status z‑value (*p* = 0.048), word list learning round 3 raw value (*p* = 0.016) and z‑value (*p* = 0.017).

In the group “mild dementia”, word list learning round 2 z‑value (*p* = 0.040), word list recognition discrimination raw value (*p* = 0.025) and z‑value (*p* = 0.016), trail making A z‑value (*p* = 0.017) and trail making B z‑value (*p* = 0.044) showed a significant correlation in each case.

Mini Mental Status z‑value (*p* = 0.025) also correlated in the group of “severe dementia”.

The results show a high standard deviation. This reflects the heterogeneous group of subjects and the broad group boundaries.

### Metabolic parameters

Table 2 shows the results of the measurements of the metabolic parameters and the respective reference ranges.

**Table 2:** Metabolic parameters

Parameter	Reference range	Group A		Group B		*p*-value
		Median	SEM	Median	SEM	
Vitamin B12 BL (pg/ml)	197–866	485.65	30.76	498.80	44.50	0.364
Vitamin B12 EP (pg/ml)		407.00	27.28	576.80	43.52	0.016
Ferritin BL (ng/ml)	13–400	97.85	18.94	89.21	21.50	0.717
Ferritin EP (ng/ml)		89.99	21.50	107.70	27.31	0.338
Folic acid BL (ng/ml)	3.1–17.5	5.48	0.88	6.11	1.06	0.238
Folic acid EP (ng/ml)		3.69	0.92	4.44	1.18	0.197
TSH BL (µU/ml)	0.27–4.2	1.32	0.87	1.37	0.25	0.856
TSH EP (µU/ml)		1.28	1.00	1.42	0.27	0.862

*BL* baseline, *EP* endpoint, *SEM* standard error of the mean, *TSH* thyroid stimulating hormone

Only vitamin B12 showed a significant difference between the two groups (*p* = 0.016). This means that in groups A and B different values in the participant’s sera were found. The median vitamin B12 concentration in group A decreased from 485.65 pg/ml to 407.00 pg/ml (*p* = 0.016) from baseline to endpoint.

No significant correlation between spermidine concentration and clinical chemical parameters was found. The results showed a significant negative correlation between vitamin B12 and “verbal fluid” (*p* = 0.002) and a significant positive correlation between folic acid and “Boston naming test” (0.017).

## Discussion

The results clearly show that oral spermidine intake significantly correlates with the improvement of cognitive performance and therefore has a positive effect on it. In both dose groups an improved CERAD score was observed after 3 months. The cohort that received the higher dosage of spermidine (group A) showed an improvement in the groups of participants with mild and moderate dementia. Statistical analysis revealed a significant correlation between the intake of spermidine and the improvement in cognitive performance. In comparison, group B showed consistency or a decrease in cognitive performance.

At the beginning it was assumed that higher cognitive performance is associated with increasing spermidine concentration, as a similar study at the Charitè showed that spermidine supplementation increases memory performance [17]. Gupta et al. also confirmed an increase in memory performance in fruit flies with higher spermidine content [7]; however, the expectation that this case occurred before the intervention was refuted in the analysis of results.

Positive correlations were found in the group classified as “no dementia”, i.e. cognitive performance increased with an increase in serum spermidine content. Since the average age of this group was the lowest, this could be a possible explanation for the result and could mean that early intervention of spermidine, starting with 60 years of age should be advised. It must be borne in mind that the memory tests are strongly dependent on the daily condition and the current motivation of the test persons. It should be also noted that the ELISA only measures the concentration of spermidine in the serum. The intracellular spermidine content was not determined. This depends on various factors, such as endogenous biosynthesis, catabolism and excretion via the kidneys. The composition of the intestinal flora is also said to influence the concentration of polyamines in humans [6].

Spermidine concentration and cognitive performance partially correlate, but in different ways. A positive correlation was found in the group without dementia. In the other groups, significant negative correlations predominated in certain cases. These cases were included in the group “mild dementia” word list learning round 2 z‑value, word list recognition discrimination raw value and z‑value, trail making A and trail making B z‑value. In the group “severe dementia” Mini Mental status z‑value and in the group “Total” word list recognizing discrimination raw value and z‑value are among them.

The statistical evaluation of the clinical chemistry parameters did not show a significant correlation between the spermidine concentration and the metabolic performance of the thyroid gland, ferritin, vitamin B12 and folic acid concentrations; however, the follow-up showed a difference in vitamin B12 concentrations in group A (*p* = 0.016). This could mean that the regular intake of spermidine can cause a decrease of vitamin B12 in the organism. The research group around Koshin Adachi published that vitamin B12 deficiency can play a role in neuronal degeneration due to a disorder of polyamine concentrations such as spermidine. Male rats were fed a vitamin B12-deficient diet or a control diet for 20 weeks and the concentrations of spermidine, spermine and putrescine, all from ornithine catabolism, were determined; however, in combination with the results obtained here, this assertion is not related to the findings from this dementia study, as group A—with the significant drop in vitamin B12—recorded a simultaneous increase in spermidine. In contrast, the Adachi study found a drop in spermidine with a vitamin B12-deficient diet. Nevertheless, it is to be emphasized that a study with human participants shows a more representative collective in contrast to experimental animals [18]. Since proton pump inhibitors (PPI) may cause a decrease of vitamin B12, we checked whether PPI intake could be a cause for vitamin B12 decrease. In fact, most persons were under PPI treatment.

In summary, we found an increase of the spermidine level in the higher dosage group from 41.65 ng/ml to 60 ng/ml (*p* < 0.001). On the contrary, the spermidine level in group B was consistent during the study. The medians in this group were always around 40 ng/ml (*p* < 0.106).

The most substantial improvement in test performance for the group with higher spermidine substitution was found in the group of subjects with mild dementia with an increase of 2.23 (*p* = 0.026) in the Mini Mental test. The improvement by more than 2 points is way beyond all available antidementia treatments so far. In a comparable study over the same period, the results were not as promising [17]. In our opinion, these differences are due to the different dosages of spermidine intake. Our group A received 2.75 times more spermidine as the intervention group in the previously quoted publication. Group B received 1.6 times more and we could not demonstrate any significant effects here either.

Based on our results, we see great therapeutic potential in spermidine supplementation in older adults at risk of dementia; however, further studies are needed, also to check whether the shown positive effect of spermidine on the cognitive function is specific or non-specific.

## Limitations

Finally, it should be noted that the aim of this paper was to show a possible positive effect of oral spermidine supplementation. The high dosage spermidine group results are far better than the achieved effects of currently available antidementia drugs. Although the effect is less in the lower dose group, a placebo effect cannot be completely excluded. Therefore, a follow-up study with a duration of 1 year is planned.

The proven correlation between oral spermidine intake and blood plasma levels needs further investigation also. In the present study, an intake with other foods cannot be excluded, although all residents from one nursing home received the same menus.
